# Supplementary material for: Vasoactive neuropeptide dysregulation: A novel mechanism of microvascular dysfunction in vascular cognitive impairment
Source: Alzheimers Dement. 2025 Nov 21;21(11):e70925. doi: 10.1002/alz.70925 (PMC12635772; doi:10.1002/alz.70925)
Supplement: Supplementary file 2 — Supporting Information [file ALZ-21-e70925-s001.docx]

**Vasoactive Neuropeptide Dysregulation: A Novel Mechanism of Microvascular Dysfunction in Vascular Cognitive Impairment**

Willians Tambo^1,2,3^, Keren Powell^1,3^, Steven Wadolowski^1,3^, Prashin Unadkat^1,4^, Eric H. Chang^3^, Christopher LeDoux^1,5^, Daniel Sciubba^3,4^, Ping Wang^2,6^, Patrica Huerta^2,7^, Chunyan Li^1,2,3,4,8*^

^1^Translational Brain Research Laboratory, The Feinstein Institutes for Medical Research, Manhasset, NY, 11030, USA.

^2^Elmezzi Graduate School of Molecular Medicine at Northwell Health, Manhasset, NY, 11030, USA

^3^Institute of Bioelectronic Medicine, The Feinstein Institutes for Medical Research, Manhasset, NY, 11030, USA

^4^Department of Neurosurgery, North Shore University Hospital at Northwell Health, Manhasset, NY 11030, USA.

^5^Department of Biology, Hofstra University, Hempstead, NY, 11030, USA

^6^Center for Immunology and Inflammation, Feinstein Institutes for Medical Research, Manhasset, NY

^7^Laboratory of Immune and Neural Networks, The Feinstein Institutes for Medical Research, Manhasset, NY, USA.

^8^Department of Neurosurgery, Zucker School of Medicine at Hofstra/Northwell, Hempstead, NY, USA

**Corresponding Author:** Chunyan Li, PhD

* The Feinstein Institutes for Medical Research, 350 Community Drive, Manhasset, NY, 11030

Phone: 1-516-562-1078

Email: cli11@northwell.edu.


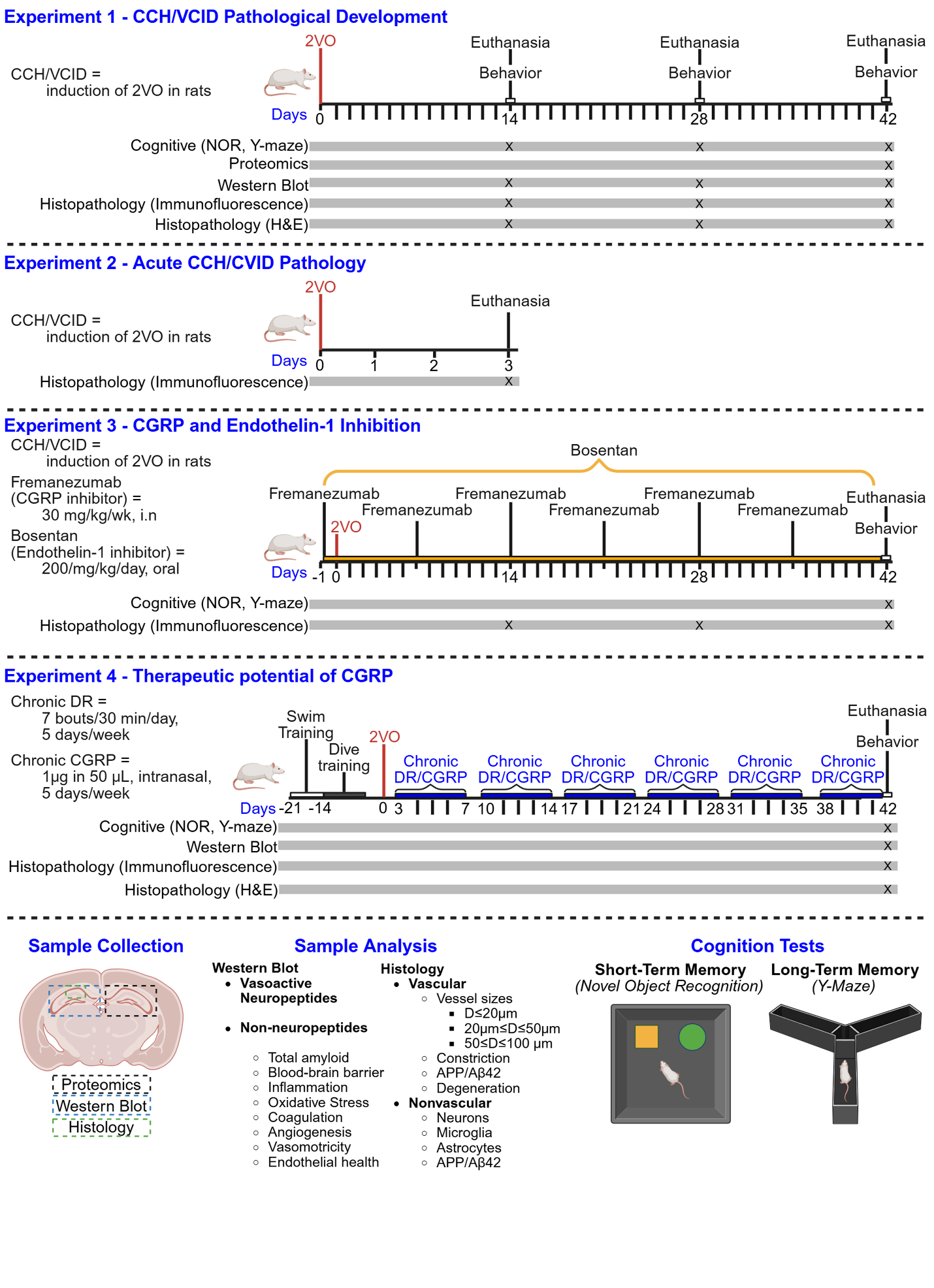


**Supplementary Figure 1. Experimental design and methodological timeline.** Schematic overview of protocols for four experiments. Experiment 1: CCH progression. Rats were evaluated at 2, 4, and 6 weeks post-2VO, with cognitive testing immediately prior to euthanasia at each time point. Neurocognitive performance was assessed using novel object recognition (NOR) and Y-maze spontaneous alternation. Pathology was comprehensively profiled, including neuropeptide and non-peptidergic biomarkers, microvascular structural integrity, and indices of parenchymal injury. Analyses integrated proteomics, Western blotting, and quantitative immunofluorescence microscopy. Experiment 2: Acute CCH pathology. Rats were examined 3 days post-2VO for select histopathological markers. Experiment 3: Inhibition of CGRP and endothelin-1. Rats were assessed at 6 weeks post-2VO, with cognitive testing at 6 weeks. Animals received Fremanezumab (CGRP inhibitor; weekly) or Bosentan (endothelin receptor antagonist; daily) throughout the survival period. Experiment 4: Therapeutic CGRP augmentation in CCH/VCID. Rats were assessed at 6 weeks post-2VO, with cognitive testing at 6 weeks. CGRP was augmented via (1) exogenous intranasal administration or (2) endogenous upregulation by diving reflex stimulation. (2VO: bilateral common carotid artery occlusion; Aβ42: amyloid β42; APP: amyloid precursor protein; CGRP: calcitonin gene-related peptide; D: diameter; **p* < 0.05, ***p* < 0.01, ****p* < 0.001, *****p* < 0.0001)


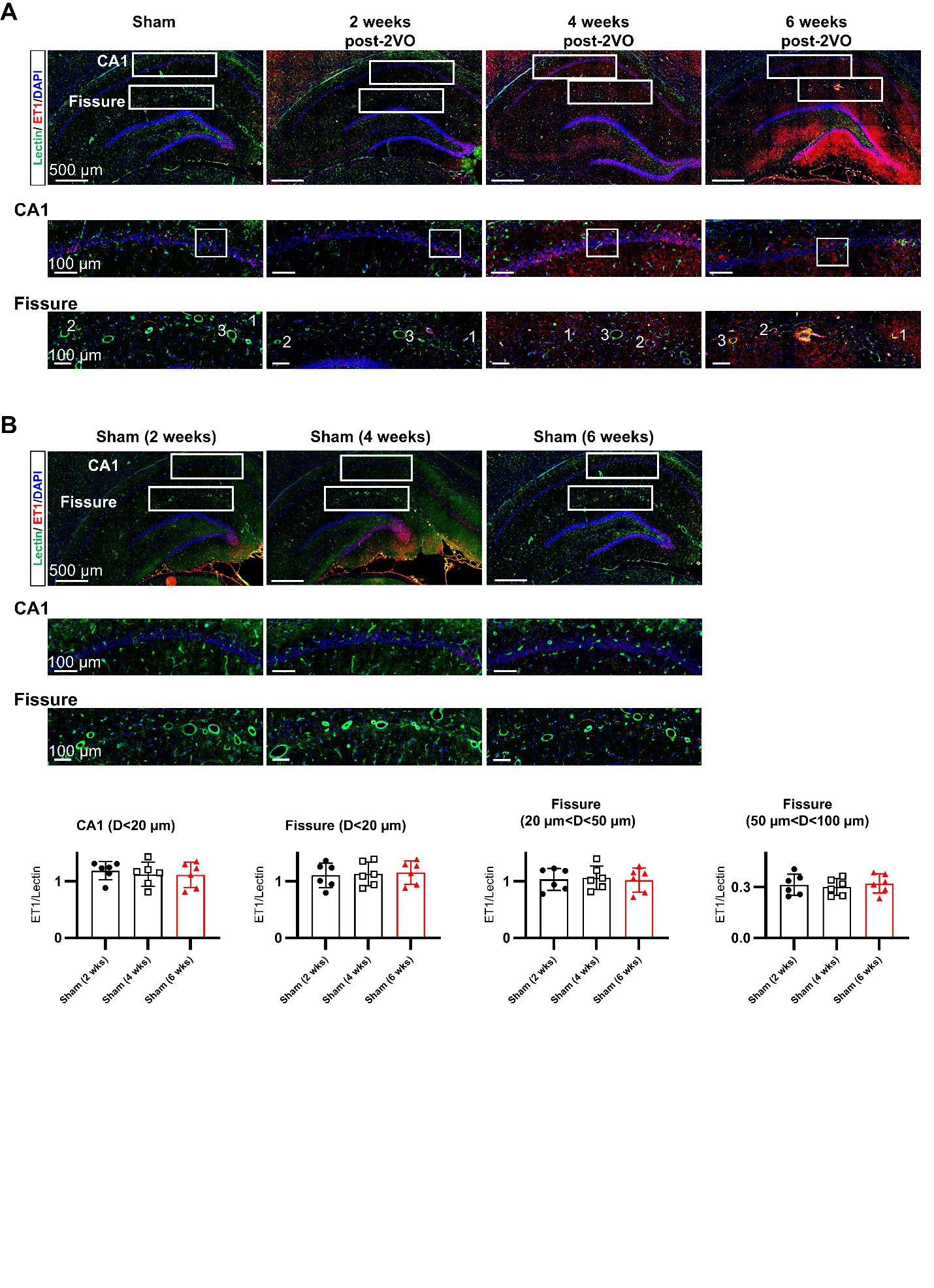
 **Supplementary Figure 2. Progressive microvascular deterioration in the hippocampus following chronic cerebral hypoperfusion.**Longitudinal evaluation of vasoconstriction in the CA1 region and hippocampal fissure of rats at 2, 4, and 6 weeks post-2VO by immunofluorescence microscopy. **(A)** Representative images showing temporal increases in ET-1 expression co-localized with lectin-labeled microvasculature in CA1 and the hippocampal fissure, indicating progressive accumulation of this vasoconstrictive peptide. **(B)** In sham animals, ET-1 immunoreactivity in CA1 and the hippocampal fissure remains unchanged across survival time points. (CCH: chronic cerebral hypoperfusion; 2VO: bilateral common carotid artery occlusion; ET-1: endothelin 1; DAPI: 4',6-diamidino-2-phenylindole; H&E: hematoxylin and eosin; **p* < 0.05, ***p* < 0.01, ****p* < 0.001, *****p* < 0.0001)


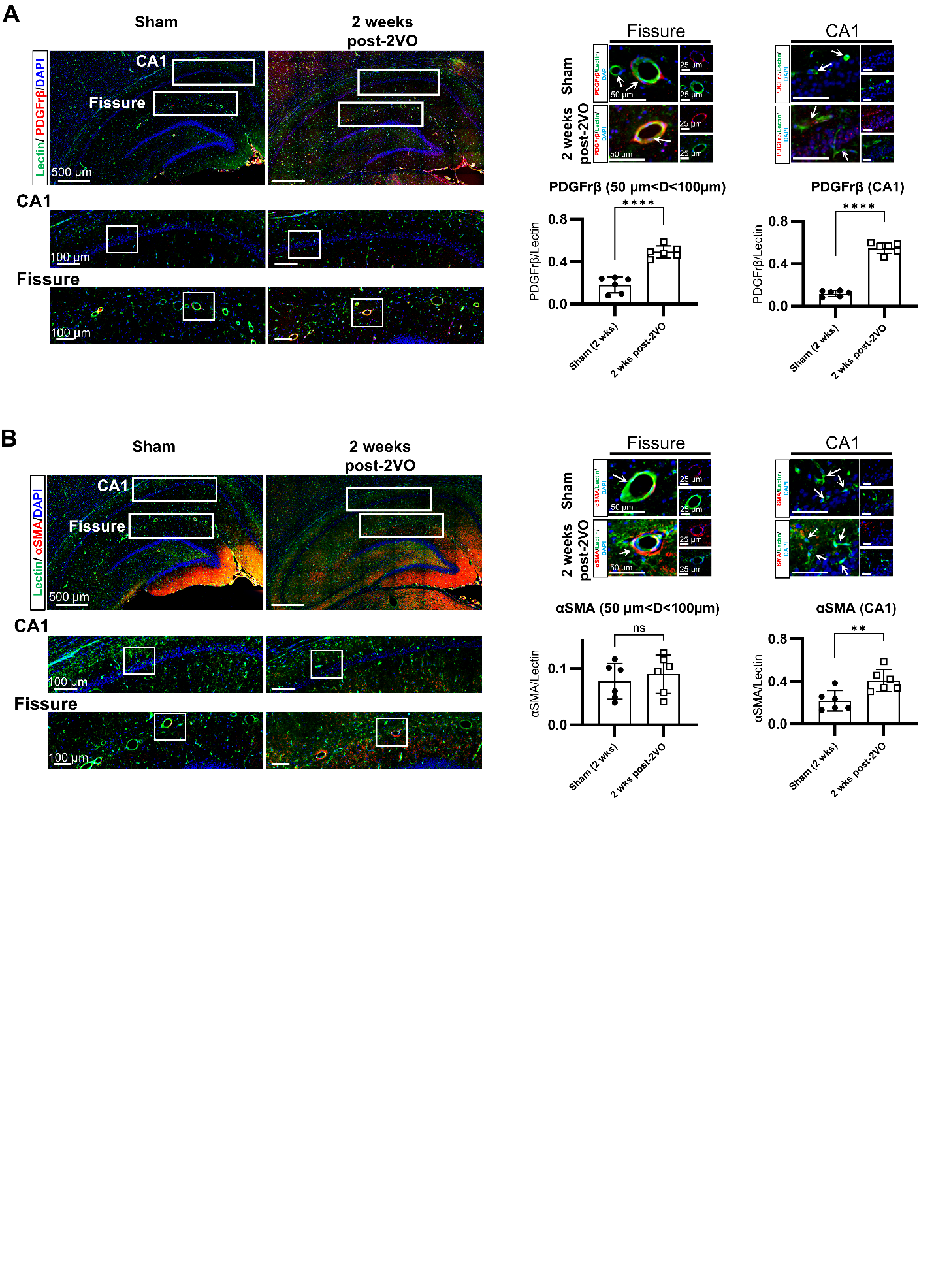
 **Supplementary Figure 3. Early hippocampal microvascular constriction following chronic cerebral hypoperfusion. Confirmation of vasoconstriction in the CA1 region and hippocampal fissure at 2 weeks post-2VO by immunofluorescence microscopy. (A)** Immunofluorescence shows pronounced PDGFRβ aggregation within vessels of the hippocampal fissure and CA1 at 2 weeks post-2VO, spanning calibers from larger fissural vessels (50 μm < D < 100 μm) to CA1 microvessels (D < 20 μm). **(B)** Robust α–smooth muscle actin (αSMA) deposition is observed in the hippocampal fissure and CA1, particularly prominent in CA1 microvessels. (Arrows indicate representative vessels analyzed.) (CCH: chronic cerebral hypoperfusion; 2VO: bilateral common carotid artery occlusion; αSMA: alpha smooth muscle actin; DAPI: 4',6-diamidino-2-phenylindole; ***p* < 0.01, *****p* < 0.0001)


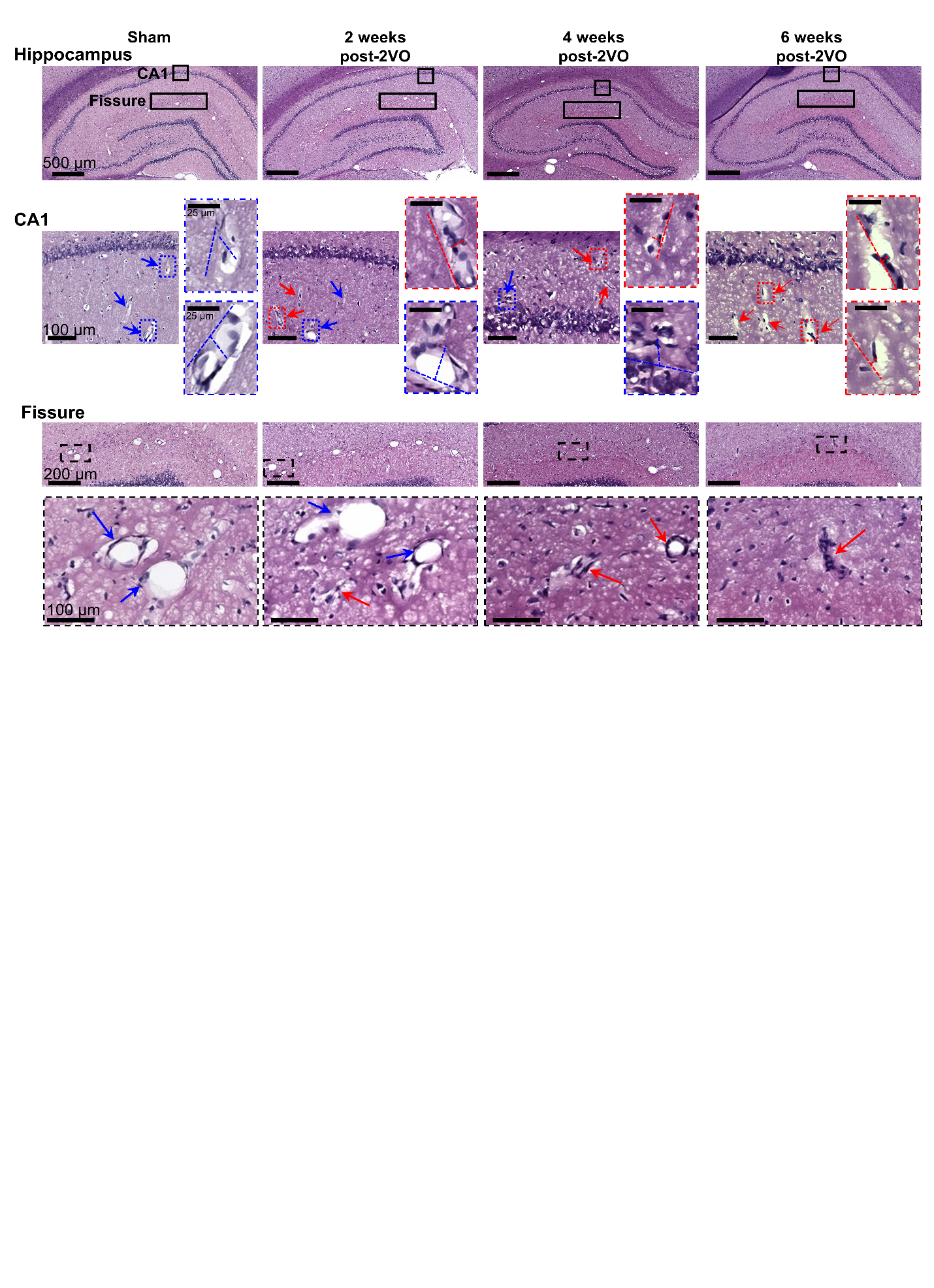


**Supplementary Figure 4. Progressive hippocampal microvascular constriction following chronic cerebral hypoperfusion.** Longitudinal histological assessment of structural changes in the CA1 region and hippocampal fissure at 2, 4, and 6 weeks post-2VO. Hematoxylin and eosin (H&E) stained sections demonstrate time-dependent microvascular constriction and collapse within CA1 and the hippocampal fissure after CCH. (arrow = collapsed vessel, red = collapsed vessel, blue = open vessel, dotted lines = vessel width and length) (CCH: chronic cerebral hypoperfusion; 2VO: bilateral common carotid artery occlusion; H&E: hematoxylin and eosin; **p* < 0.05, ***p* < 0.01, ****p* < 0.001, *****p* < 0.0001)


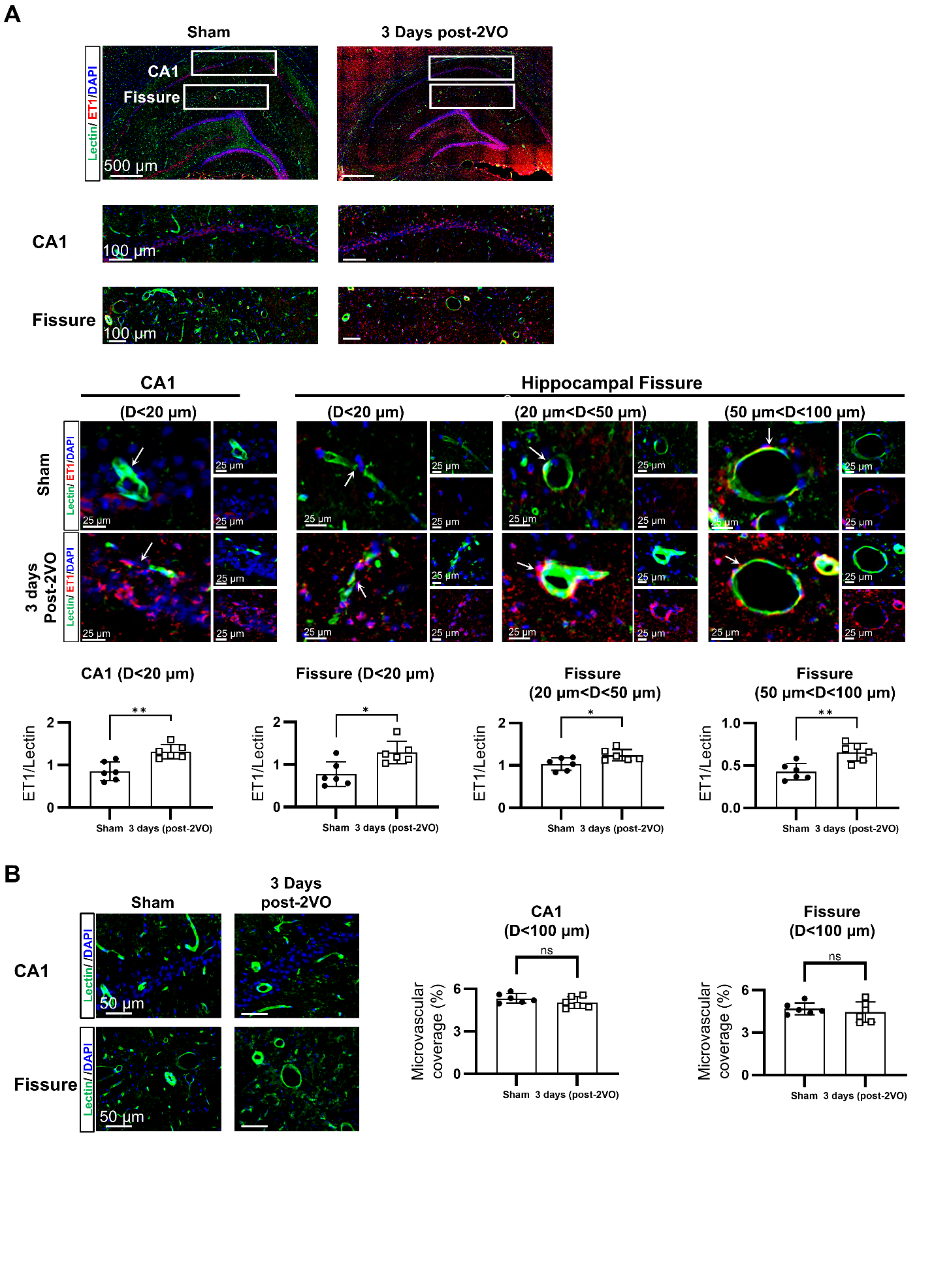
 **Supplementary Figure 5. Early hippocampal microvascular constriction precedes microvascular degeneration following chronic cerebral hypoperfusion.** Immunofluorescence assessment at 3 days post-2VO confirms acute vasoconstriction in the hippocampus. **(A)** Co-localization of endothelin-1 (ET-1) with lectin-labeled vessels in CA1 and the hippocampal fissure demonstrates pronounced microvascular constriction at the acute time point. **(B)** Lectin staining in CA1 and the hippocampal fissure shows no detectable loss of microvasculature at this early stage. (CCH: chronic cerebral hypoperfusion; 2VO: bilateral common carotid artery occlusion; ET-1: endothelin-1; DAPI: 4',6-diamidino-2-phenylindole; D: diameter; *p < 0.05, **p < 0.01)


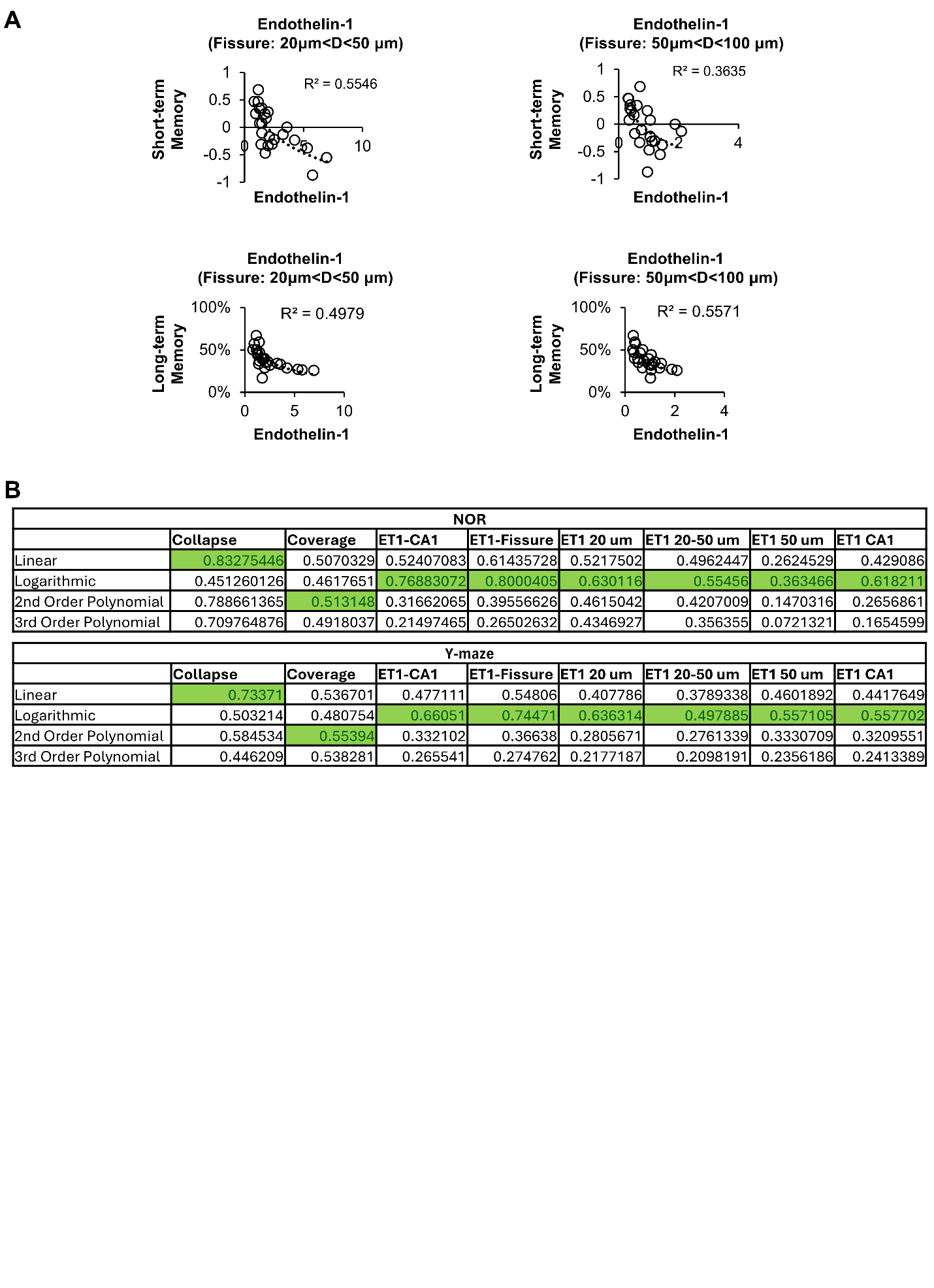
 **Supplementary Figure 6. Correlation between microvascular constriction and cognitive deterioration in chronic cerebral hypoperfusion.**Regression analyses examining the relationship between microvascular endothelin-1 immunoreactivity and cognitive performance measures in the chronic cerebral hypoperfusion model. **(A)** Quantitative assessment revealed a statistically significant fair-to-moderate negative correlation between endothelin-1 expression and memory function parameters. The strength of this correlation was contingent upon microvascular diameter, with the correlation coefficient demonstrating vessel caliber-dependent variation, suggesting differential susceptibility of microvasculature to endothelin-mediated vasoconstriction as a potential mechanism underlying cognitive impairment. **(B)** Evaluation using four regression models shows that although ET-1 levels, microvascular constriction, and degeneration are negatively associated with cognitive function, the relationships are not strictly linear, consistent with heterogeneous temporal dynamics. (ET1: endothelin-1)


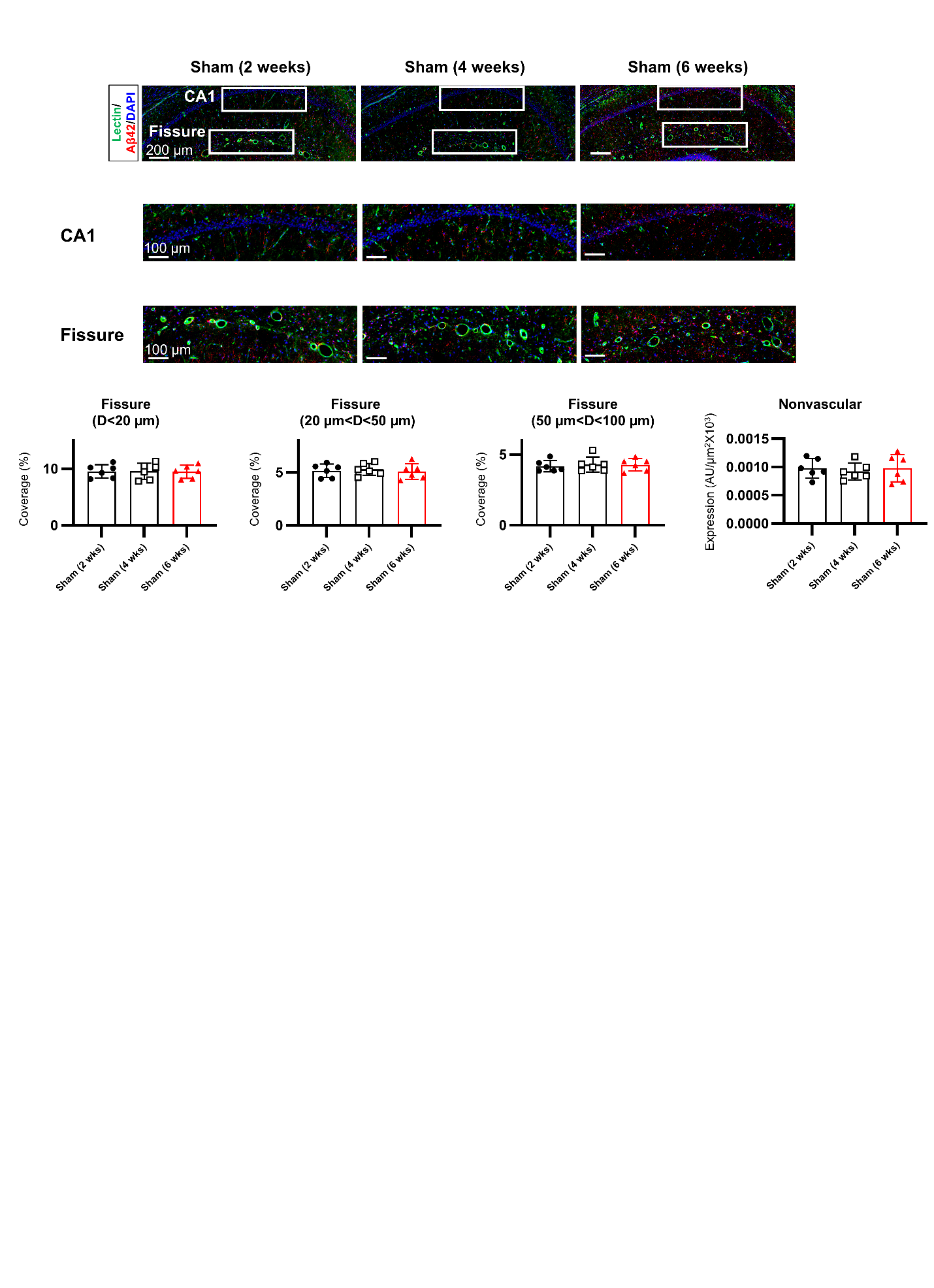
 **Supplementary Figure 7. Survival time does not influence amyloid aggregation in sham-operated animals.** Immunofluorescence imaging shows no significant Aβ42 accumulation in the CA1 region or hippocampal fissure of sham animals across all survival time points. (CCH: chronic cerebral hypoperfusion; 2VO: bilateral common carotid artery occlusion; Aβ42: amyloid β42; DAPI: 4',6-diamidino-2-phenylindole; D: diameter; *p < 0.05, **p < 0.01)


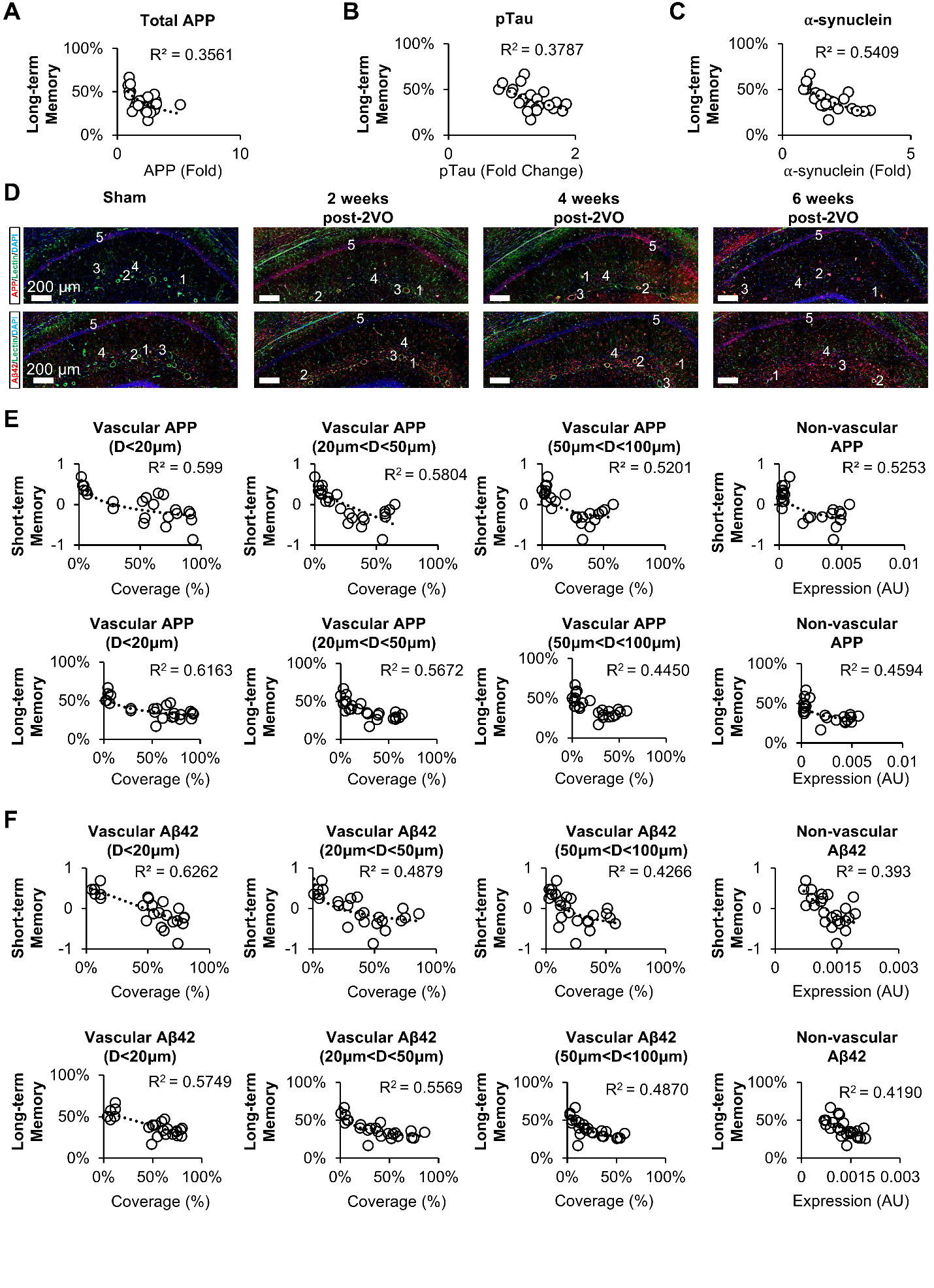
 **Supplementary Figure 8. Differential correlation of vascular-associated versus total amyloidogenic protein expression with cognitive impairment in chronic cerebral hypoperfusion. (A-C)**Regression analyses examining the relationship between total hippocampal amyloidogenic protein burden and long-term spatial memory performance in the chronic cerebral hypoperfusion (CCH) model. Quantification of total amyloid precursor protein (APP), phosphorylated tau (pTau), and α-synuclein immunoreactivity demonstrated not strong correlations with cognitive performance metrics. **(D)** Representative immunohistochemical visualization of APP and Aβ42 distribution in hippocampal tissue following CCH induction (numerical indicators correspond to panels in Figure 4D, 4E of the main manuscript). **(E-F)** Comparative correlation analyses of vascular-localized versus parenchymal APP expression with vessel diameter parameters and cognitive assessment scores. Correlation coefficients exhibited vessel caliber-dependency, with significant negative correlations observed in small-diameter microvessels and progressively diminishing correlation strength with increasing vessel diameter, culminating in non-significant associations in non-vascular parenchymal regions. These findings suggest compartment-specific contributions of amyloidogenic protein accumulation to cognitive dysfunction following CCH. (CCH: chronic cerebral hypoperfusion; APP: amyloid precursor protein; pTau: phosphorylated tau, Aβ42: amyloid β42; ET-1: endothelin 1; **p* < 0.05, ***p* < 0.01, ****p* < 0.001, *****p* < 0.0001)


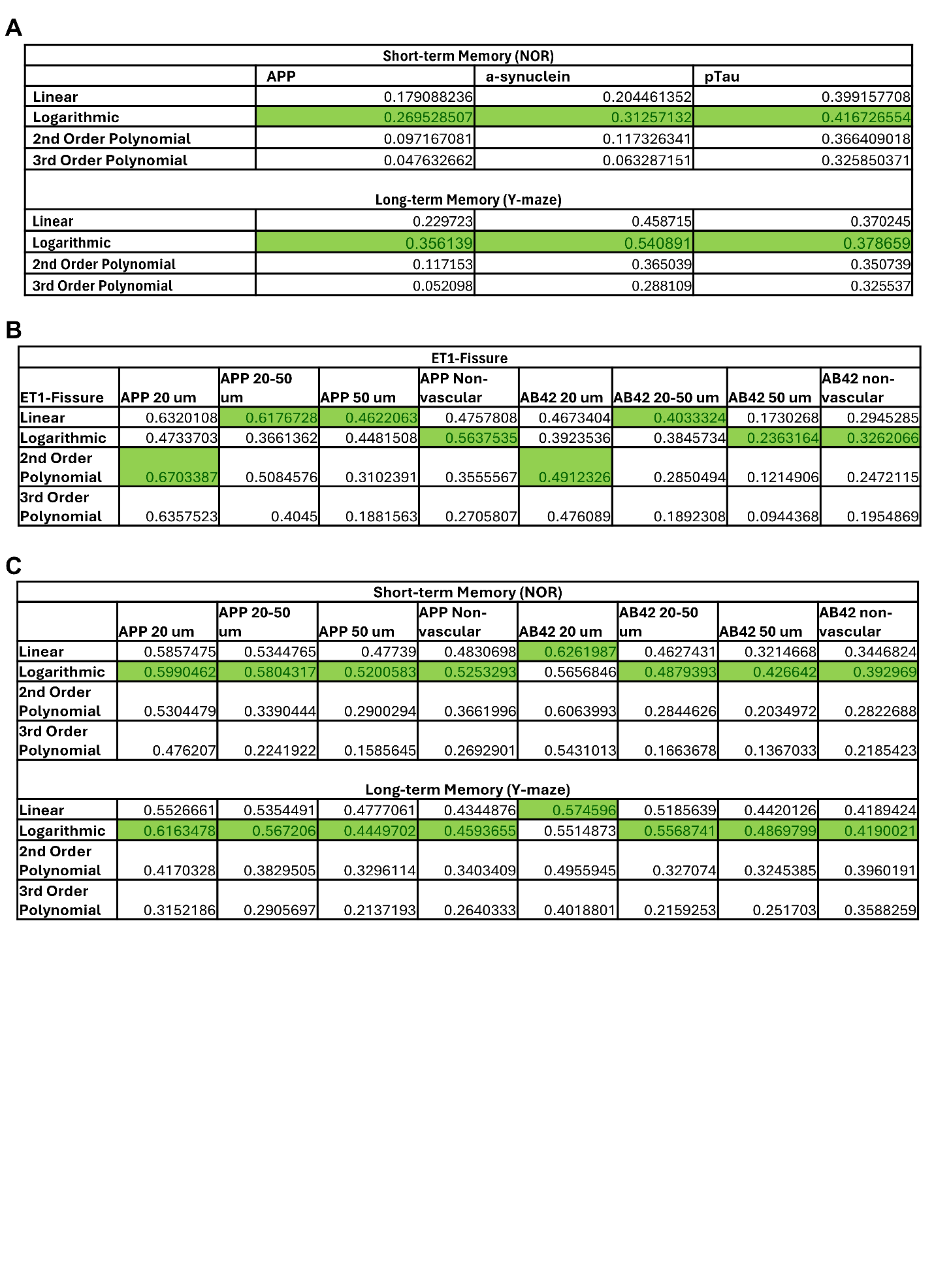
 **Supplementary Figure 9. Differential correlations between amyloidogenic protein burden and cognitive impairment in chronic cerebral hypoperfusion.** Schematic overview of four regression analyses examining relationships among **(A)** cognitive outcomes and total hippocampal amyloidogenic protein burden; **(B)** vascular and non-vascular amyloid expression and endothelin-1 levels; and **(C)** cognitive outcomes and vascular versus non-vascular amyloid expression. (CCH: chronic cerebral hypoperfusion; APP: amyloid precursor protein; pTau: phosphorylated tau, Aβ42: amyloid β42; ET1: endothelin 1; **p* < 0.05, ***p* < 0.01, ****p* < 0.001, *****p* < 0.0001)


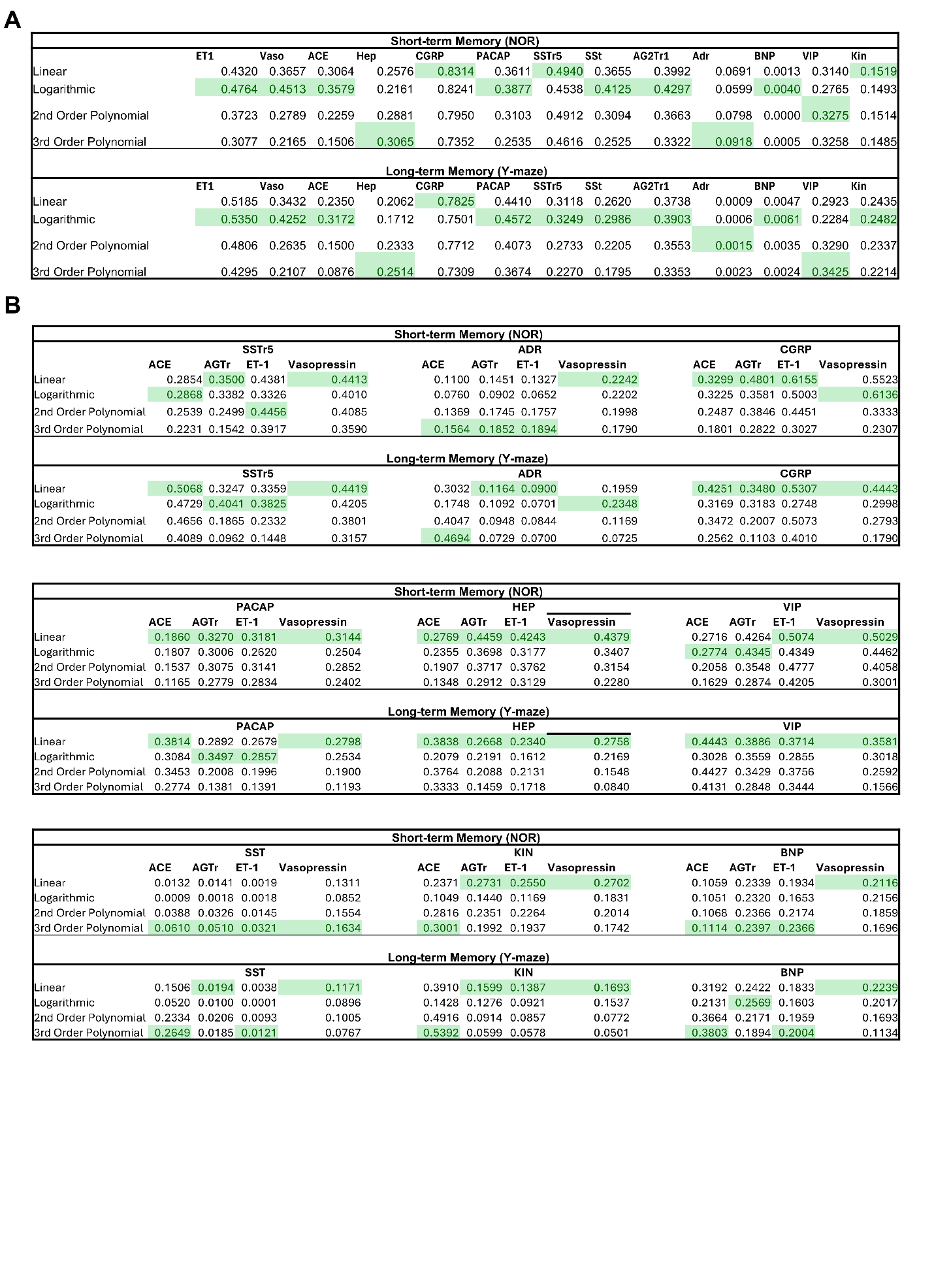
 **Supplementary Figure 10. Vasomotor neuropeptide dysregulation correlates with progressive memory decline.** Schematic summary of four regression approaches evaluating relationships between **(A)** cognitive outcomes and total vasoactive neuropeptide expression, and **(B)** cognitive outcomes and derived vasoactive neuropeptide expression ratios. The strongest correlations with cognitive impairment were observed for CGRP, the most potent vasodilator measured, and for CGRP-based ratios, followed consistently by ET-1, the most potent vasoconstrictive neuropeptide. (CCH: chronic cerebral hypoperfusion; 2VO: bilateral common carotid artery occlusion; HEP: hepcidin; SST: somatostatin; SSTr5: somatostatin receptor 5; ET-1: endothelin-1; CGRP: calcitonin gene-related peptide; Vaso: vasopressin, AT2r1: angiotensin II receptor 1; ACE: angiotensin converting enzyme; VIP: vasoactive intestinal peptide; PACAP: pituitary adenylate cyclic-activating polypeptide; ADR: adrenomedullin)


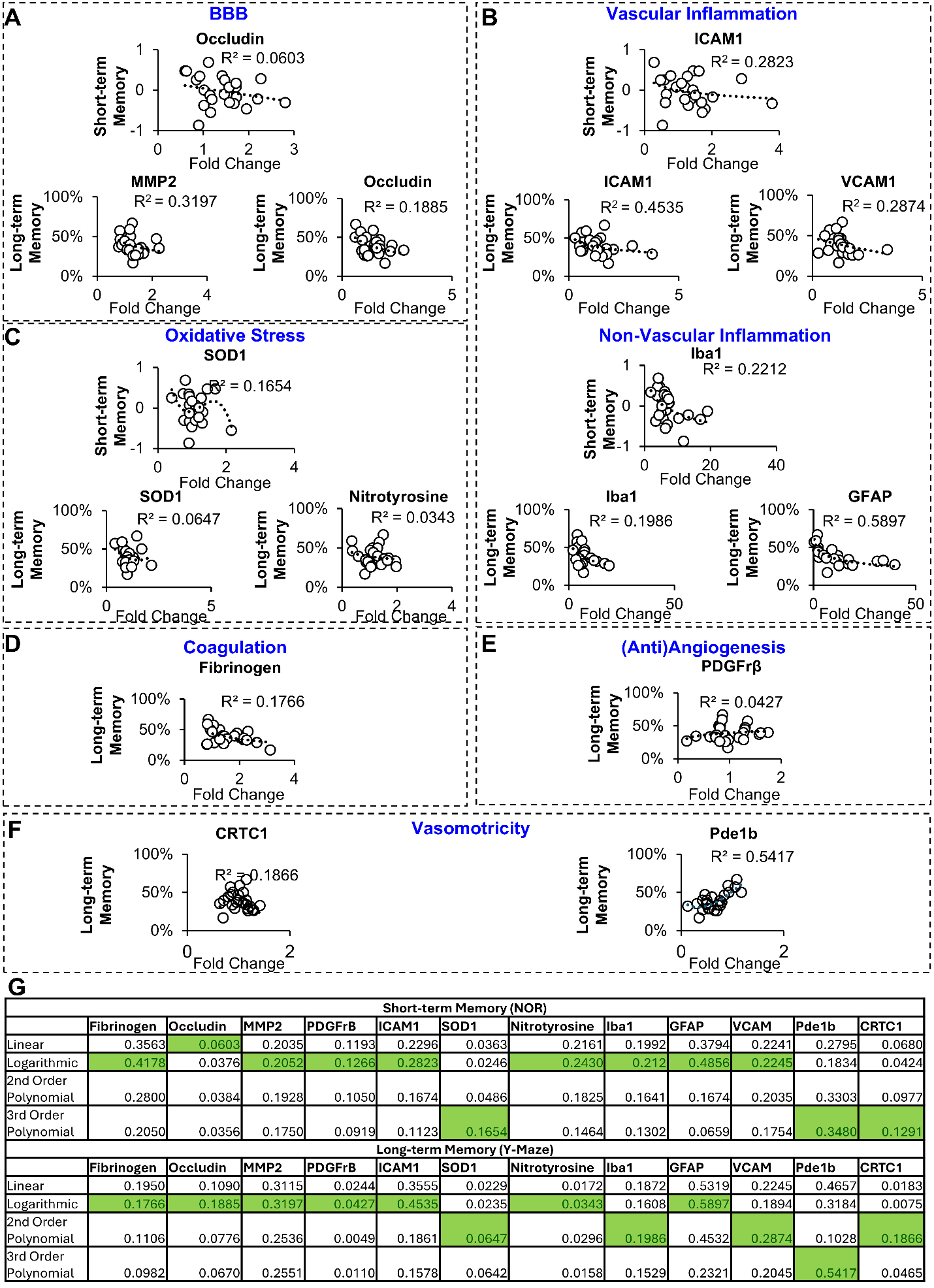
 **Supplementary Figure 11. Minimal correlation between nonvascular pathological features and cognitive dysfunction following chronic cerebral hypoperfusion.  (A-F)** Correlation analyses examining relationships between quantified nonvascular pathological indicators and cognitive performance parameters. It revealed predominantly weak correlations across multiple markers of parenchymal injury. These findings suggest that, unlike vascular pathology, nonvascular tissue alterations contribute minimally to the cognitive impairment observed in this experimental model of CCH. **(G)** Schematic summarizing four regression approaches evaluating relationships between cognitive outcomes and non-vascular pathological indicators. (CCH: chronic cerebral hypoperfusion; 2VO: bilateral common carotid artery occlusion; MMP2: matrix metalloproteinase 2, BBB: blood brain barrier; ICAM1: intercellular adhesion molecule 1; VCAM1: vascular cell adhesion molecule 1; SOD: superoxide dismutase; NT: nitrotyrosine; PDGFrβ: platelet derived growth factor receptor β; Pde1b: Phosphodiesterase 1B; CRTC1: CREB-regulated transcription coactivator 1; Iba1: ionized calcium binding adaptor molecule 1; GFAP: glial fibrillary acidic protein)


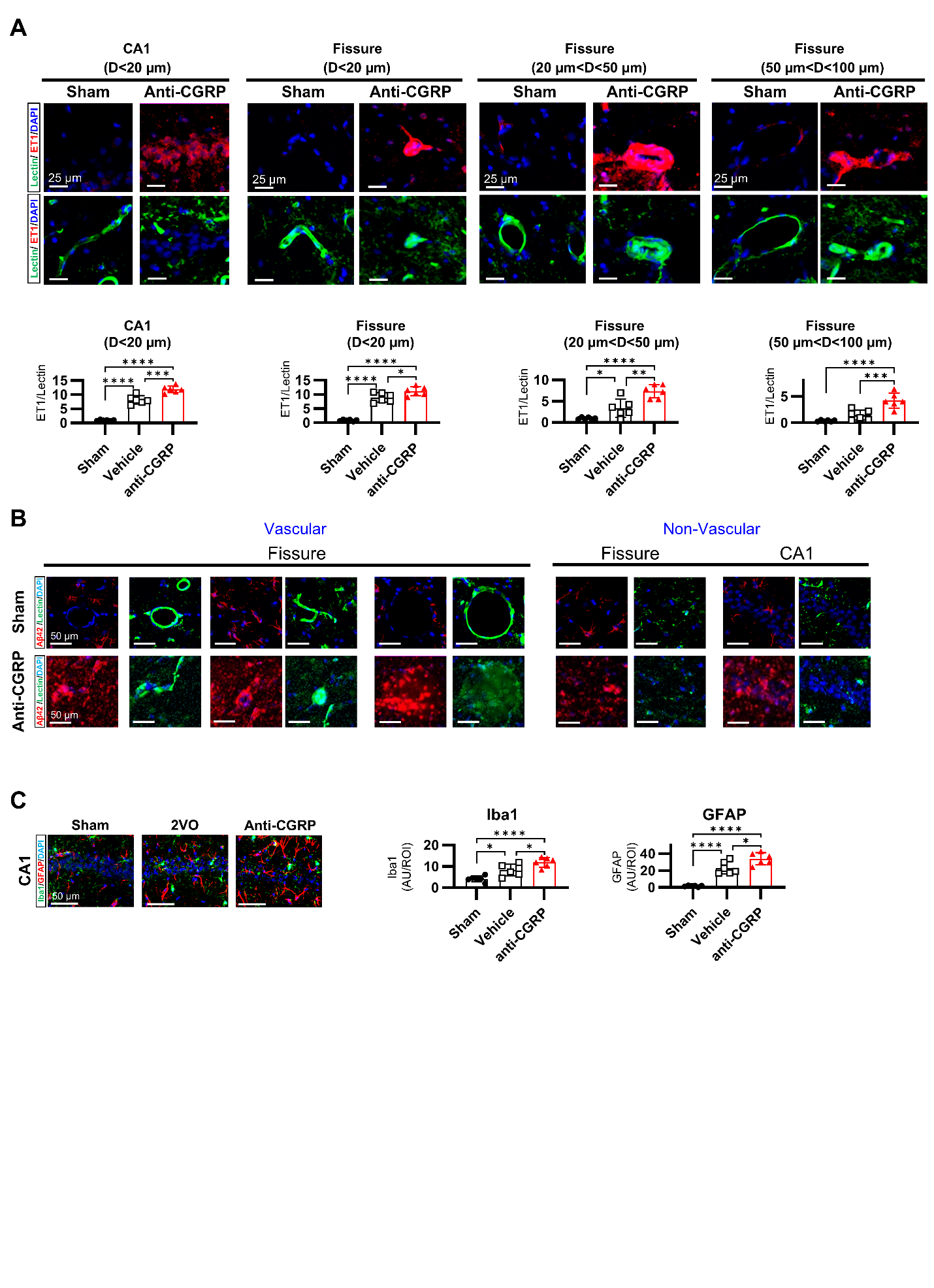
 **Supplementary Figure 12. CGRP inhibition exacerbates pathology in chronic cerebral hypoperfusion.** Immunofluorescence reveals worsening vasoconstriction, amyloid deposition, and inflammatory signals with CGRP blockade. **(A)** Single-channel imaging of ET-1 and lectin shows increased microvascular ET-1 burden following further CGRP reduction in CCH, most pronounced in larger-caliber microvessels that were relatively spared with CCH-induced CGRP downregulation alone. **(B)** Single-channel imaging of Aβ42 and lectin demonstrates increased Aβ42 accumulation within both vascular and extravascular compartments upon CGRP inhibition in CCH. (CCH: chronic cerebral hypoperfusion; 2VO: bilateral common carotid artery occlusion; Aβ42: amyloid β42; ET1: endothelin-1; CGRP: calcitonin gene-related peptide DAPI: 4',6-diamidino-2-phenylindole; D: diameter; *p < 0.05, **p < 0.01)


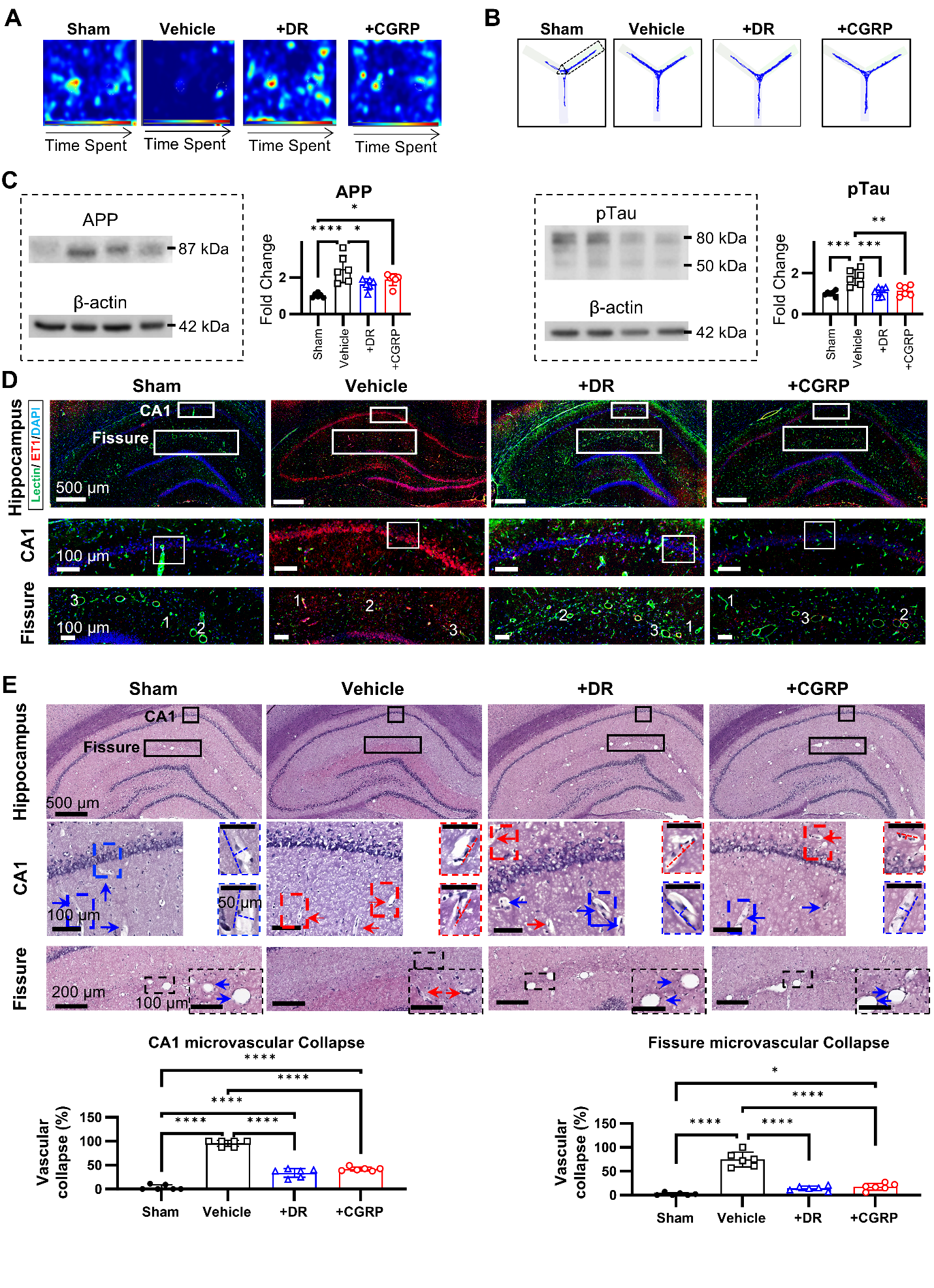


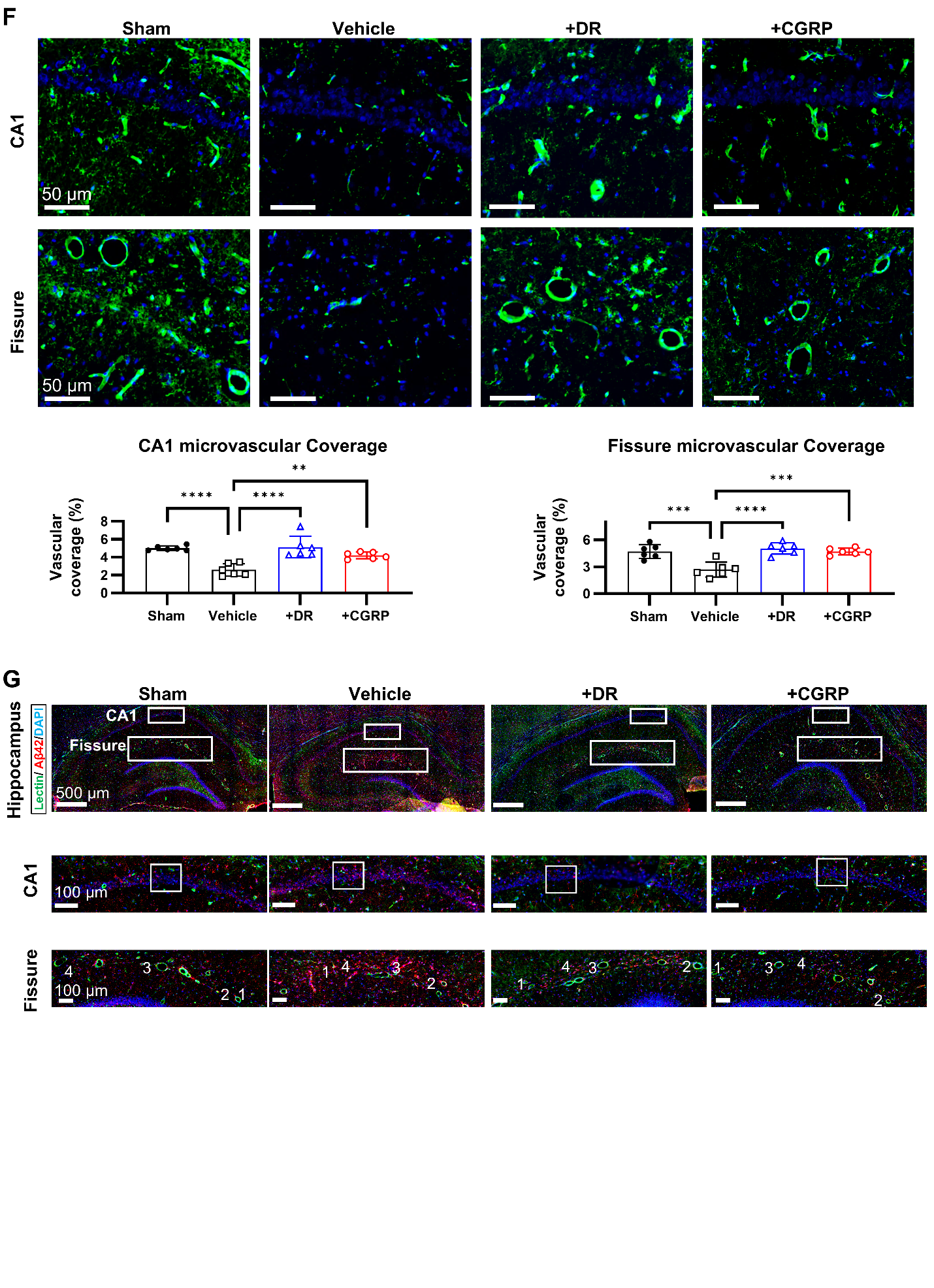

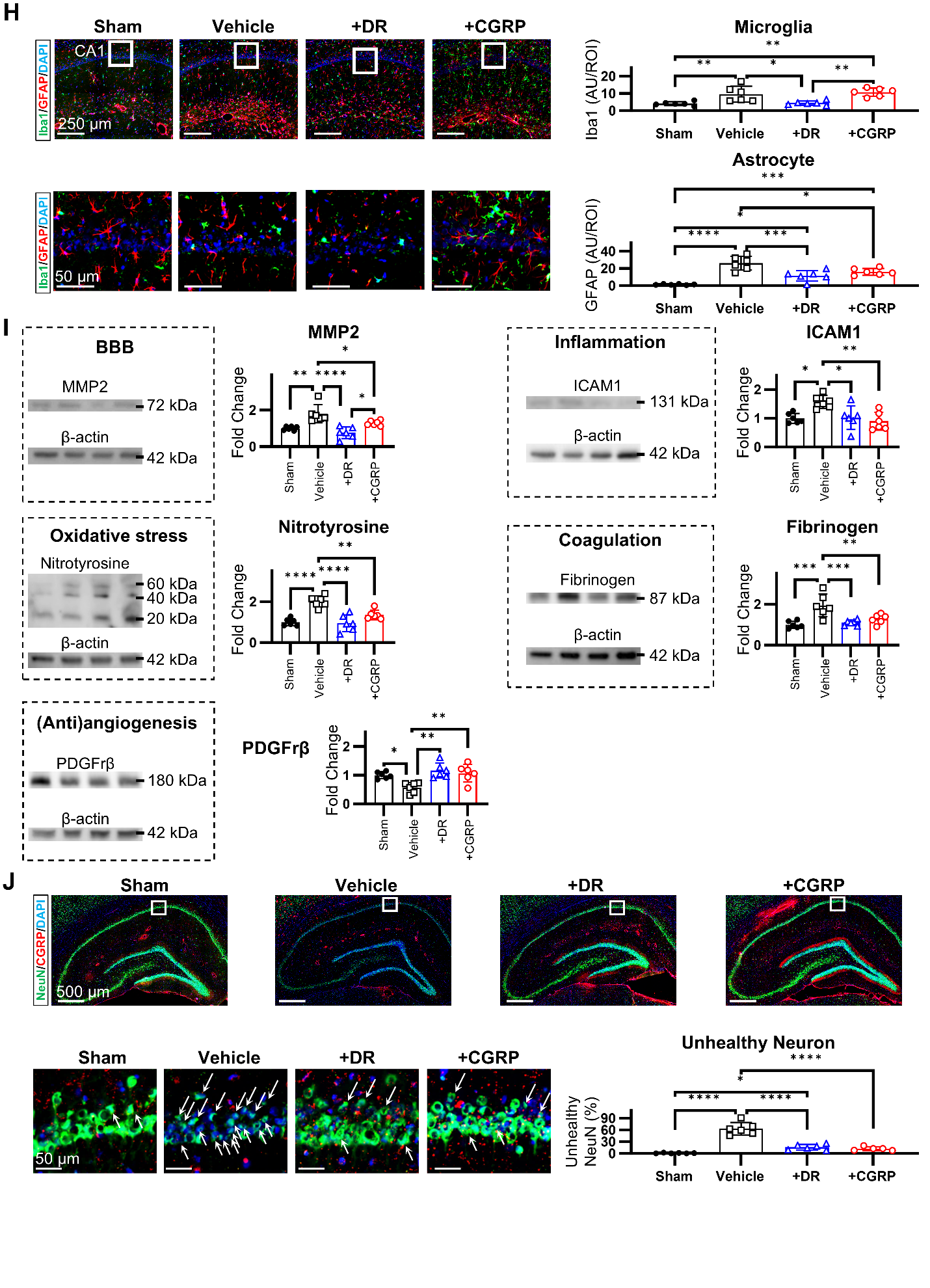
 **Supplementary Figure 13. CGRP supplementation mitigates cognitive dysfunction through attenuation of microvascular and non-vascular pathologies. (A-B)** CGRP supplementation significantly ameliorated both short-term and long-term memory deficits at 6 weeks following bilateral common carotid artery occlusion (2VO). **(C)** Densitometric analysis demonstrated that endogenous and exogenous CGRP modulation significantly downregulated amyloid precursor protein (APP) and phosphorylated Tau (pTau) expression at 6 weeks post-2VO. **(D)** Immunofluorescence imaging revealed that both endogenous and exogenous CGRP supplementation attenuated endothelin-1 (ET-1) expression at 6 weeks post-2VO. **(E)** Semi-quantitative analysis demonstrated that CGRP supplementation significantly mitigated microvascular collapse within the CA1 and fissure regions of the hippocampus. **(F)** Quantitative immunofluorescence analysis showed that endogenous and exogenous CGRP supplementation significantly increased microvascular coverage. **(G)** Immunofluorescence imaging demonstrated that CGRP administration decreased Aβ42 expression throughout the hippocampus. **(H)** Quantitative immunofluorescence analysis revealed that endogenous and exogenous CGRP supplementation decreased both Iba1 and GFAP expression at 6 weeks post-2VO, indicating reduced neuroinflammation. **(I)** Densitometric analysis indicated that CGRP modulation mediated the amelioration of both vascular and non-vascular pathogenesis. **(J)** Quantitative immunofluorescence assessment demonstrated that CGRP supplementation significantly improved neuronal viability in the CA1 subregion of the hippocampus. (CCH: chronic cerebral hypoperfusion; 2VO: bilateral common carotid artery occlusion; MMP2: matrix metalloproteinase 2, BBB: blood brain barrier; ICAM1: intercellular adhesion molecule 1; VCAM1: vascular cell adhesion molecule 1; SOD: superoxide dismutase; NT: nitrotyrosine; PDGFrβ: platelet derived growth factor receptor β; Pde1b: Phosphodiesterase 1B; CRTC1: CREB-regulated transcription coactivator 1; Iba1: ionized calcium binding adaptor molecule 1; GFAP: glial fibrillary acidic protein; **p* < 0.05, ***p* < 0.01, ****p* < 0.001, *****p* < 0.0001) (arrow = collapsed vessel, red = collapsed vessel, blue = open vessel, dotted lines = vessel width and length)


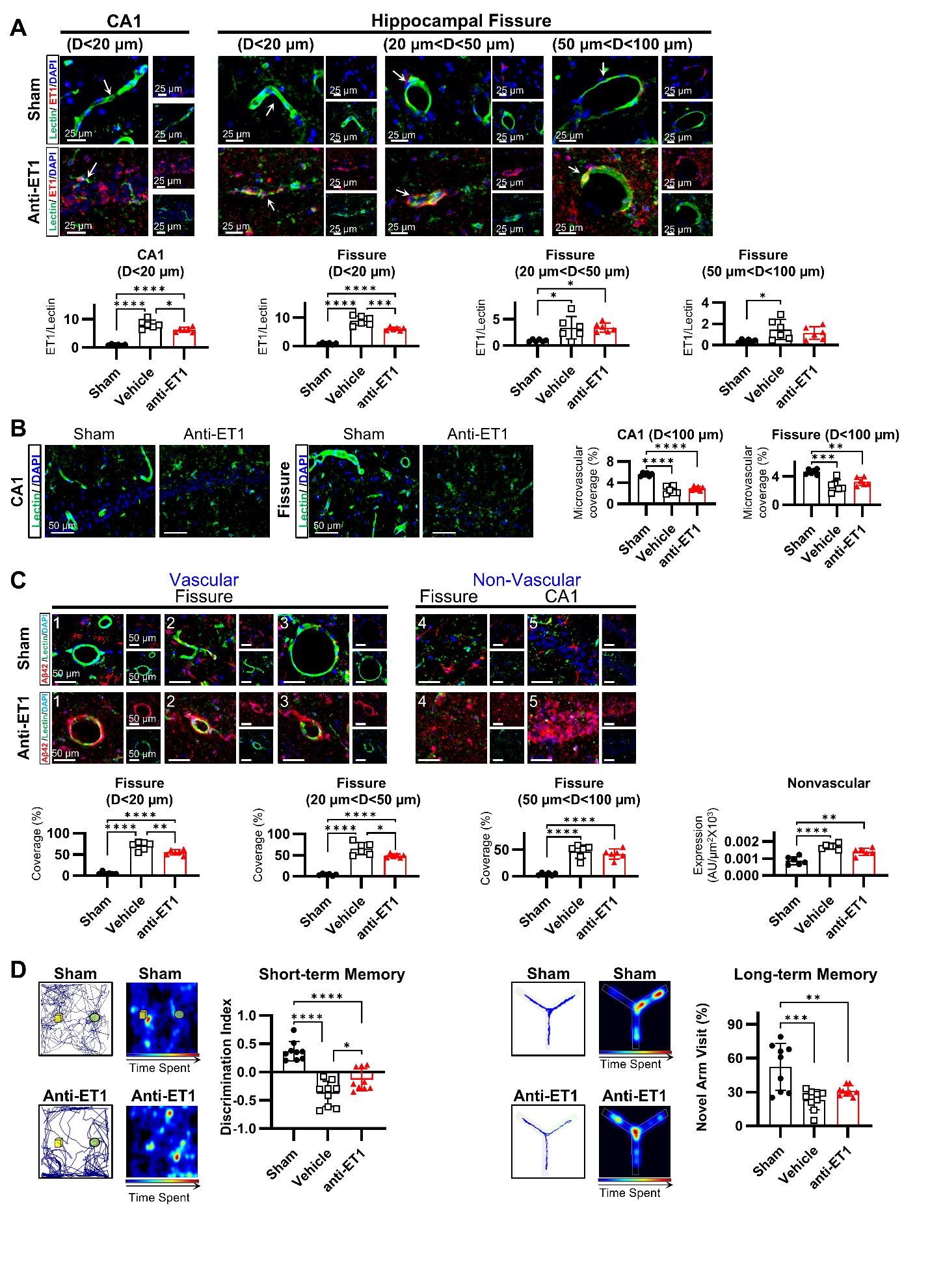
 **Supplementary Figure 14. Endothelin-1 inhibition is less effective than CGRP supplementation in attenuating CCH-associated pathology.** An integrated evaluation of vasoconstriction, vascular degeneration, amyloid burden, and cognitive outcomes indicates a comparatively reduced therapeutic impact of ET-1 blockade. **(A)** Immunofluorescence for endothelin-1 and lectin shows only modest attenuation of microvascular constriction and intravascular ET-1 signal with ET-1 inhibition. **(B)** Quantitative immunofluorescence demonstrates no improvement in microvascular coverage. **(C)** ET-1 inhibition lowers Aβ42 levels in microvessels and larger fissural vessels (50 μm < D < 100 μm), but not in mid-caliber vessels (20 μm < D < 50 μm), with overall effects smaller than those achieved by CGRP supplementation. **(D)** Behaviorally, ET-1 inhibition significantly improves short-term, but not long-term, memory at 6 weeks post-CCH. (CCH: chronic cerebral hypoperfusion; 2VO: bilateral common carotid artery occlusion; Aβ42: amyloid β42; ET1: endothelin-1; CGRP: calcitonin gene-related peptide; DAPI: 4',6-diamidino-2-phenylindole; D: diameter; *p < 0.05, **p < 0.01)
